# Leveraging artificial intelligence for perioperative cancer risk assessment of oral potentially malignant disorders

**DOI:** 10.1097/JS9.0000000000000979

**Published:** 2023-12-04

**Authors:** John Adeoye, Yu-Xiong Su

**Affiliations:** Division of Oral and Maxillofacial Surgery, Faculty of Dentistry, University of Hong Kong, Hong Kong SAR, People’s Republic of China

**Keywords:** artificial intelligence, oral cancer, oral potentially malignant disorders, risk prediction, surgical excision

## Abstract

Oral potentially malignant disorders (OPMDs) are mucosal conditions with an inherent disposition to develop oral squamous cell carcinoma. Surgical management is the most preferred strategy to prevent malignant transformation in OPMDs, and surgical approaches to treatment include conventional scalpel excision, laser surgery, cryotherapy, and photodynamic therapy. However, in reality, since all patients with OPMDs will not develop oral squamous cell carcinoma in their lifetime, there is a need to stratify patients according to their risk of malignant transformation to streamline surgical intervention for patients with the highest risks. Artificial intelligence (AI) has the potential to integrate disparate factors influencing malignant transformation for robust, precise, and personalized cancer risk stratification of OPMD patients than current methods to determine the need for surgical resection, excision, or re-excision. Therefore, this article overviews existing AI models and tools, presents a clinical implementation pathway, and discusses necessary refinements to aid the clinical application of AI-based platforms for cancer risk stratification of OPMDs in surgical practice.

## Introduction

Highlights

- Oral potentially malignant disorders (OPMDs) have an increased risk to develop oral squamous cell carcinoma.
- Cancer risk assessment is crucial to determine the need for surgical, excision, or re-excision among patients with OPMDs.
- Oral epithelial dysplasia grading is commonly used for cancer risk assessment but has several limitations.
- Artificial intelligence (AI) can help overcome the limitations of the dysplasia grading systems and provide better risk stratification for patients with OPMDs.
- Recommendations to promote the actualization and meaningful clinical application of AI in perioperative cancer risk assessment of OPMDs are provided.

### Background

Oral potentially malignant disorders (OPMDs) are common mucosal conditions involving the lip and oral mucosa with an inherent disposition for malignant transformation to oral squamous cell carcinoma (OSCC) or infrequently verrucous carcinoma^1,2^. The 2020 WHO classification of the disorders comprises 11 disease entities such as leukoplakia, proliferative verrucous leukoplakia, erythroplakia, oral lichen planus, actinic cheilitis, and dyskeratosis congenita^1^. Surgical intervention, which may include resection, excision, or ablation, represents the most common and preferred strategy to prevent OSCC development in many OPMDs^3^. Current modalities of surgical intervention include traditional scalpel or electrocautery excision, laser excision/ablation, cryotherapy, and photodynamic therapy with the nonconventional methods offering some benefit in alleviating postoperative pain and edema as well as improving wound healing provided there is no impedance to the histologic assessment of lesions for definitive diagnosis^3–6^.

Cancer risk assessment is important to the effective surgical management of OPMDs. As all patients with OPMDs do not develop OSCC in their lifetime, preoperative risk stratification/prediction of individual lesions is required to determine or support the rationale for surgical intervention as opposed to an observational approach that is indicated for low-grade lesions^3,7^. In addition, the findings of a randomized controlled trial and different observational studies have shown that OSCC may still occur among patients who undergo surgical excision^8,9^. Thus, underscoring the need for postoperative cancer risk assessment to promote timely intervention for recurrent or residual lesions and to facilitate close disease surveillance for early OSCC detection^10^. Moreover, these cancer risk assessment tools may be useful for guiding patient selection in clinical trials of surgical interventions for OPMDs^3^.

### Standard approach, problem statement, and new approach

Detection and grading of oral epithelial dysplasia (OED) upon histologic evaluation represents the most popular method for stratifying the risk of malignant transformation in OPMDs perioperatively^11^. Though useful, this method has shortfalls that have been highlighted extensively. First, OSCC development may be observed among OPMD patients without OED resulting in poor treatment selection for these patients in surgical centers^12^. Second, different OED grading systems exist, which makes clinical reporting ambiguous and introduces subjectivity into cancer risk assessment^11,13,14^. Last, there is no consensus on the accuracy or precision of the OED grading method for treatment selection in the management of OPMDs^11,15^. With the availability of other risk factors for oral cancer development such as demography, risk habits, lesion size, anatomic sites, clinical appearance, comorbidities, and molecular markers, an approach that integrates other factors to optimize the predictive ability of dysplasia is warranted in line with the need for precise and individualized risk stratification in OPMDs surgical management.

Artificial intelligence (AI) is an advanced contemporary technique for constructing risk prediction models in clinical practice^16,17^. The approach, which is more streamlined to predictive ability than other statistical methods, involves using algorithms capable of deciphering patterns from data without explicit instructions and generalizing to new patient data based on learned patterns in a process known as machine learning. Of note, AI has the potential to be incorporated into surgical practice for virtual planning, interactive intraoperative guidance, surgical robotics, and clinical decision-support systems^18–20^. Therefore, this article overviews the application of AI models/tools/platforms for the perioperative cancer risk prediction of patients with OPMDs drawing from our experience with the construction and application of AI methods in this field. Additionally, we present a detailed pathway to clinical implementation and discuss refinements to aid the meaningful application of AI models in the cancer risk assessment of patients with OPMDs before and after surgical excision.

## How does AI improve perioperative cancer risk prediction of OPMDs?

AI simply refers to the use of computers or computer-controlled devices to simulate human intelligence^21^. Even with this definition, the current stage of AI implementation in healthcare is narrow or weak, which denotes that the platforms are goal-oriented and largely able to perform singular tasks^22^. In the perioperative cancer risk assessment of OPMDs, AI tools offer two key advantages compared to the current practice of dysplasia grading. First, AI models can optimize the predictive ability of dysplasia presence and grading by integrating this feature with other predisposing factors such as demographic information (age and sex), risk habits (tobacco smoking/chewing, heavy alcohol consumption, and areca nut chewing), clinical characteristics (lesion size, anatomic site, color, texture, and induration status), comorbidities (Charlson comorbidity index, hepatitis infection, and autoimmune diseases), and molecular markers (loss of heterozygosity, salivary, plasma, and tissue-based biomarkers) to determine patients that may require surgical excision or may be at risk of re-excision^7,23,24^. The benefit of this integration results in an objective and holistic risk profiling of patients being considered for surgical intervention and may assist in planning disease surveillance regimens following surgery. Moreover, the feasibility of constructing and applying this category of AI models in surgical centers stems from the availability of predictive parameters in structured formats from electronic/manual health records of patients with OPMDs. Second, AI models are capable of extracting features from unstructured clinical investigation records of patients with OPMDs (including clinical photographs, imaging, and whole-slide images) and detecting patterns from these features to provide predicted probabilities of malignant transformation that may objectively assist surgeons in patient decision making^25,26^.

## Implementation pathway for AI platforms in OPMD cancer risk prediction

**Figure 1:**
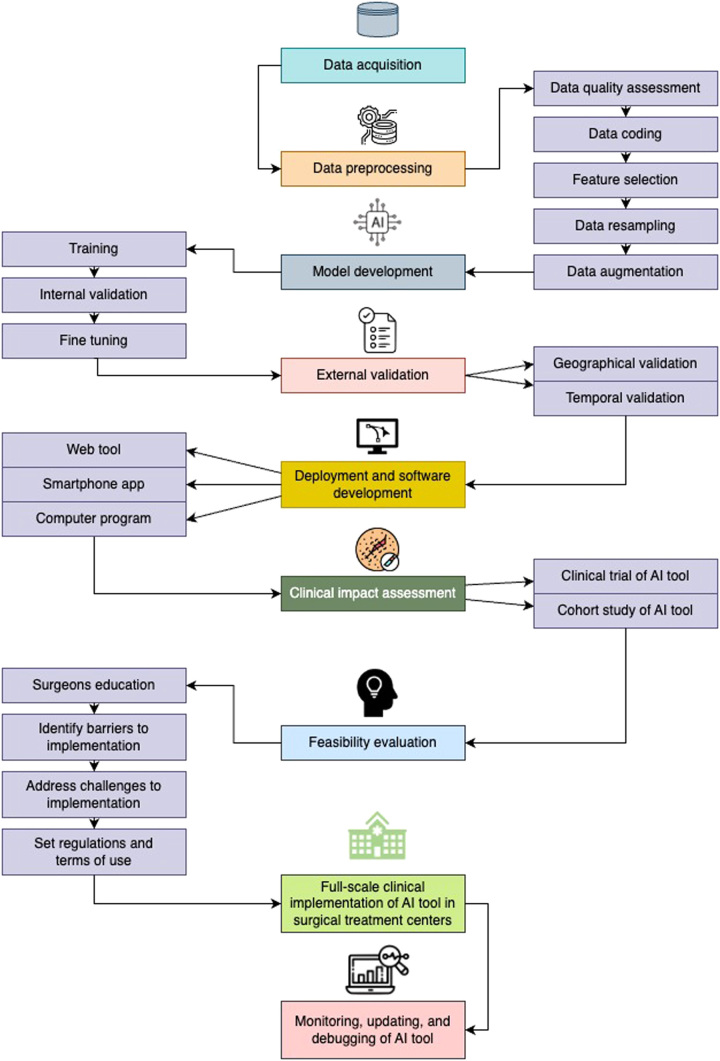
Proposed implementation pathway for artificial intelligence (AI)-based models or platforms for cancer risk assessment in the surgical management of oral potentially malignant disorders.

The pathway to implementing AI platforms in the cancer risk assessment of OPMDs to determine candidates for surgical excision is presented in Figure 1 and starts with data acquisition and preparation for machine learning^27^. Clinical data to be employed in AI model development for this task is likely to be retrospective as the time to malignant transformation of OPMDs is variable and may be lengthy if prospective data collection is planned^3,10^. Furthermore, data for AI tool development may be structured (such as cohort information obtainable from health records as predictive risk factors) or unstructured including imaging, photographs, whole-slide images, blots, and spectral images^28^. Nonetheless, the type of clinical data available is likely to guide the choice of AI model to be constructed.

Data collected are then preprocessed mainly by following the stages highlighted in Figure 1 before the AI model is developed. The quality of datasets for model construction is assessed to provide some expectations on the potential performance of the AI model in line with available data and to determine potential areas for improving data if feasible. Parameters to be evaluated are determined by the type of datasets (structured vs unstructured data) and may include data completeness, data fairness, class overlap, image resolution, label purity, data representativeness, outlier detection, feature relevance, and target class parity^28,29^. Of note, cohorts of patients with OPMDs are likely to be imbalanced (i.e. have poor class parity) since malignant transformation occurs only in a smaller subset of patients^30^. As such, class imbalance correction techniques like minority class oversampling, majority class undersampling, transfer learning, and data augmentation are deemed crucial during preprocessing of data to construct AI models for predicting malignant transformation in OPMDs^31^. For structured data, data encoding techniques such as label encoding, binary encoding, one-hot transformation, and outcome encoding may be performed to enable the use of AI algorithms that can only implement numerical labels and to improve the ability of the AI algorithms to recognize patterns for cancer risk prediction^32,33^. Additionally, selecting relevant predictive features and data resampling methods (e.g. cross-validation, bootstrapping, and jackknife resampling) are techniques that can boost the performance of AI models in cancer risk prediction and prevent overfitting during model construction, especially with low-volume datasets of OPMD cohorts^34^.

AI model development involves training and internal validation, which should be conducted with a subset of the entire OPMD patient data to enable further fine-tuning. Calibration, discriminatory performance, model stability, and interpretability of techniques should also be assessed during internal validation^35^. Afterward, external validation of the AI model should be performed using similar data of OPMD patients from other surgical centers within and outside the region of model construction (geographical) or with data from the same center from a different period (temporal) to ensure generalizability^36^. At this stage, explainability of the rationale behind the predictions of the AI-based model should also be implemented. If the AI model is found to be robust following external validation, an AI-based platform or user interface (such as a web tool or smartphone application) may be deployed with the model as the backend. It is this AI platform/tool that is then assessed for its clinical impact in either a randomized controlled trial involving patients with OPMDs or an observational study to determine whether any benefit exists in employing the tool for malignant transformation prediction^37,38^. However, since the time to observe malignant transformation or an adequate follow-up time may take several years, an observational clinical impact assessment approach such as a cohort study may be more feasible.

Two types of outcomes should be considered during the clinical impact assessment of AI platforms for perioperative malignant transformation prediction of OPMDs. One outcome should assess the accuracy of novel AI platforms in comparison to dysplasia presence and grading systems. Additionally, the second outcome should compare the rate or proportion of OSCC development when AI-based risk stratification is used to select patients for surgical excision against when dysplasia presence and grading is used for cancer risk prediction.

Upon the confirmation of the potential clinical impact, actions to evaluate and improve the feasibility of the AI platform in surgical practices should be taken. Barriers to clinical application should also be identified at this stage including organizational, socioeconomic, ethical, and legal/regulatory concerns about the AI-based tool^39,40^. Adapting the surgical treatment protocol of patients with OPMDs towards a more digitized system and addressing a potential increase in patients’ treatment time represent key organizational changes to be implemented before the clinical application of AI-based models. As AI applications may be considered novel in OPMD management, educating oral and maxillofacial, ENT, head and neck, and plastic surgeons (especially young surgeons) on the method and benefits of applying the AI tool in daily practice is required^41^. The additional cost of purchasing and maintaining AI-based tools, lack of patient knowledge about AI mechanisms and benefits, and distrust in AI clinical judgments by patients are relevant potential financial and social barriers to the implementation of AI-based tools for perioperative cancer risk stratification among patients with OPMDs^42^.

Ethical and legal issues that may hamper the implementation of AI-based tools for perioperative cancer risk stratification may include concerns about the accuracy and net benefit of the intelligent tool, the safety and interpretability of predictions in surgical practice, data and algorithm transparency, dependency of clinicians on AI-based tool (potentially among young surgeons), queries on the need for informed consent before the use of intelligent platforms, data privacy, patient data protection, undefined terms of use, bias of AI-based model among patients in remote regions or ethnic minorities, and concerns on who takes responsibility for treatment decisions made based on wrong predictions^43–45^. Of note, surgeons should be aware that some form of agreement should be reached with patients after detailed explanations of the risks and benefits of the AI method before clinical application^39,45,46^. Since the accuracy and generalizability of the AI models may be improved with multicenter development and validation, federated learning may be considered in place of central learning to prevent breaching patient privacy during the use of secondary data for model construction^47,48^. Multicenter data for model construction should promote data inclusivity by including patients with OPMDs across different ethnicities and socioeconomic groups especially those from low or lower-middle-income countries^39,45^. Likewise, AI-based platforms should also be fashioned to not retain patient information or clinical investigation records, which are provided as input variables for estimating malignant transformation probability among patients with OPMDs.

Full-scale implementation in surgical centers for clinical decision support among OPMD patients may be considered once these barriers have been addressed and detailed regulations have been established. Monitoring, fixing technical issues, and model updating when new data becomes available should continue indefinitely to ensure the constant application of the AI tool in the surgical management of patients with OPMDs.

## Existing AI models/platforms for perioperative cancer risk prediction of OPMDs

A systematic review conducted in 2021 to determine the accuracy of AI algorithms in classifying oral cancer outcomes found that only a few models were developed to stratify malignant transformation risk in OMPDs and no AI-based platform for potential clinical application was available^49^. However, since this study, more promising AI models and tools have emerged. An updated search of the electronic databases in July 2023 (PubMed, Scopus, EMBASE, Cochrane Library, LILACS, SciELO, PscychINFO, and Web of Science) using similar search criteria to the previous study^49^ found a total of 10 studies reporting AI models for perioperative risk stratification of OPMDs (Table 1)^50–59^. Overall, most of the AI models available for perioperative oral cancer risk assessment have largely focused on the feasibility of AI architectures for clinical prediction rather than the development of platforms with obvious benefits to surgical practice.

**Table 1:** AI models for predicting oral cancer risk in oral potentially malignant disorders by year of development.

Author	Year	Study location(s)	OPMD subtype	Treatment strategies	OPMD cohort size (% events)	AI algorithms	Data employed	Type of validation	Model accuracy	Software / deployment	Application in surgical centers	Timing of application in surgical centers	Refs
Baik *et al.*	2014	Canada	Unspecified. OED used for patient selection	Not specified	71 (50.7)	Random forest	Slide images	Internal	SE: 78% SP: 71% ACC: 75%	Yes (algorithm)	No	N/A	^50^
Liu *et al.*	2017	China	Leukoplakia	Not specified	110 (6.4)	Random forest	DNA image cytometry	Internal	SE: 57% SP: 85% ACC: 84%	No	No	N/A	^51^
Shams *et al.*	2017	USA	Leukoplakia	Not specified	86 (40.7)	Deep neural network	Gene expression profile	Internal	SE: 98% SP: 94% ACC: 97%	No	No	N/A	^52^
Wang *et al.*	2020	China	Leukoplakia, erythroplakia, and lichenoid disease	Surgical excision and medical treatment	101 (12.9)	Random forest	Medical records and noninvasive tests (Autofluorescence and vital dye staining)	External	SE: 62% SP: 75% ACC: 73%	Yes (web tool)	Yes	Preoperative	^53^
Adeoye *et al.*	2021	Hong Kong SAR and UK	Leukoplakia and lichenoid disease	Traditional excision, laser surgery, medical treatment, and observation	1098 (8.9)	DeepSurv	Electronic health records	External	C-index: 0.82 IBS: 0.18	Yes (web tool)	Yes	Preoperative and postoperative	^54^
Ellis *et al.*	2022	UK	Leukoplakia	Surgical excision, observation	17 (58.8)	Principal component analysis - Linear discriminant analysis (PCA-LDA)	Hyperspectral images	Internal	SE: 79% SP: 76% ACC: 77%	No	No	N/A	^55^
Ferrer-Sánchez *et al.*	2022	Spain	Leukoplakia	Conventional and laser surgery	261 (13.4)	U-net and CNN	Clinical photographs	Internal	SE: 100% SP: 69% ACC: 74%	No	No	N/A	^56^
Wu *et al.*	2022	USA	Leukoplakia and lichenoid disease	Unspecified	2192 (34)	Gradient boosting	Electronic health records	Internal	SE: 74% SP: 84% ACC: 80%	No	No	N/A	^57^
Adeoye *et al.*	2023	Hong Kong SAR, UK, and Nigeria	Leukoplakia and lichenoid disease	Traditional excision, laser surgery, medical treatment, and observation	1187 (8.3)	Random forest and Light GBM	Electronic health records	External	SE: 100% SP: 88% ACC: 90%	Yes (web tool)	Yes	Preoperative and postoperative	^58^
Cai *et al.*	2023	China	Leukoplakia	Surgical excision, Photodynamic therapy, Medical treatment	759 (11.9)	ResNet50 and LightGBM	Whole-slide images	External	SE: 100% SP: 67%	No	No	N/A	^59^

ACC, accuracy; SE, sensitivity; SP, specificity.

### Data sources and quality

Data used to construct AI models for perioperative cancer risk assessment of OPMDs were chiefly structured data of clinicopathological characteristics and treatment information of patients that were obtained from health records rather than histology slide images, DNA image cytometry, clinical photographs, gene expression profile data, or hyperspectral images (Table 1). Private datasets were also utilized in all but one study that used a public gene expression dataset^52^. Also, only two cohorts obtained prospectively were used to construct AI models for cancer risk assessment in OPMDs^51,53^. Data was pooled from two or more treatment centers during model construction in five studies^53,54,57–59^ while others used data from a single treatment center (Table 1). Of these, only two AI models were constructed using multinational cohorts from Hong Kong, the UK, and Nigeria^54,58^. The sample size of cohorts used for model construction ranged from 17 to 2192 patients (Table 1) with only four models involving data from at least 500 patients^54,57–59^. Malignant transformation of patients with OPMDs within the cohorts also ranged from 6.4 to 58.8%. For datasets with an outcome class ratio (cancer vs no cancer) above 1 to 2^51,53,54,56,58,59^, only three AI models were constructed after adjusting for class imbalance using techniques such as data augmentation, synthetic minority oversampling technique (SMOTE), adaptive synthetic technique (ADASYN), and class weight optimization^56,58,59^.

### AI algorithms and model construction

Supervised machine learning classifiers were the algorithm of choice for binary risk stratification of malignant transformation among patients with OPMDs. Of note, conventional machine learning techniques (especially random forest and other ensemble learning methods) were mostly used to construct models for perioperative cancer risk assessment of OPMDs. Neural networks were used for the construction of four AI-based models two of which used a multilayer perceptron with backpropagation architecture^52,54^ while another two predictive models were based on convolutional neural networks^56,59^. Only four of the AI models^53,54,58,59^ have undergone external (geographical) validation with others being internally validated only. Likewise, the AI models had accuracy, sensitivity, and specificity values of 73– 97%, 57–100%, and 67–94%, respectively (Table 1). Of note, five AI models (which corresponded to the most recent models) employed feature relevance and visualization techniques to explain the rationale for cancer risk prediction including color coding^55^, local interpretable model-agnostic explanations (LIME)^56^, Shapley additive explanations^57,58^, and Gradient-weighted class activation mapping (Grad-CAM)^59^.

### AI models by specific OPMD subtypes

All cohorts comprised patients with oral leukoplakia, although a few models utilized cohorts that also included patients with oral lichenoid disease and erythroplakia but not exclusively^53,54,57,58^. No AI-based models were found to predict (perioperative) cancer risk among patients with proliferative verrucous leukoplakia, actinic cheilitis, reverse smokers’ palate, oral lupus erythematosus, oral graft versus host disease, oral erythroplakia, oral lichen planus, and oral lichenoid lesions exclusively. Of note, performance metrics for the AI-based models that incorporated disparate OPMD entities were reported for all OPMDs included rather than for the individual clinical subtypes^53,54,57,58^. As such, the validity of the intelligent models in perioperative cancer risk assessment for distinct OPMD subtypes other than oral leukoplakia remains obscure. For the five AI-based models for cancer risk prediction in oral leukoplakia only^51,52,55,56,59^, their accuracy was from 74 to 97%, sensitivity from 57 to 100%, and specificity from 67 to 94% (Table 1).

### Current AI-based platforms for perioperative cancer risk assessment in OPMDs

Three models have been deployed as AI-based web platforms to allow for further verification of their performance and to aid their potential application in surgical practice. Predictive performance and AI model specifications for these intelligent platforms specifically are also provided in Table 1.

One AI-based web tool with the DeepSurv feed-forward multilayer neural network as the backend (available at http://oralcancerai.hku.hk) requires 26 input variables that are often available from electronic health records in surgical centers to generate a time-to-event cancer risk probability curve for up to 270 months following diagnosis^54^. Moreover, this AI web platform may be employed to determine cancer risk probabilities before treatment planning to advise on whether surgical intervention is needed and after surgical management to determine the potential for re-excision, the need for periodic field biopsy, and disease surveillance strategy. To support single timepoint cancer risk stratification of OPMDs, a supplementary model was also developed based on a similar concept, validated externally, and deployed as a web tool to provide binary prediction of malignant transformation risk status and a predicted probability of cancer occurrence (available at https://opmd-predict-facdent-hku.herokuapp.com)^58^.

The AI-based web tool by Wang *et al.* ^53^ based on the random forest algorithm (available at http://web.opmd-risk.com), which was originally developed to delineate the grade of OPMDs, was also observed to correlate with the risk of malignant transformation following prospective external validation prompting its potential application in surgical practice. The AI tool requires 10 mandatory inputs (including two adjunctive tests i.e. tissue autofluorescence imaging and toluidine blue staining) and 10 optional inputs to generate a binary oral cancer risk status (high risk vs low risk) of patients with OPMDs. However, the timing of application for this web tool in the surgical management of OPMDs may be limited to preoperative assessment since no input variable considered treatment strategy and the OPMD cohort used for development also included those that had only pharmacologic intervention.

## Considerations to aid clinical implementation of AI-based tools in perioperative cancer risk assessment of OPMDs

### Application criteria of AI-based platforms in the surgical management of OPMDs should be specified and followed

To encourage the full implementation of AI-based methods for perioperative oral cancer risk stratification among OPMD patients, there should be detailed and unambiguous criteria to guide application. This is lacking for the existing AI platforms as the requirements and settings of their application can only be inferred from the studies. Specifically, the preferred period to apply the AI tools whether preoperatively during the assessment of the need for surgical excision or postoperatively to monitor oral cancer risk should be specified as early as during AI model construction. This is important as some predictors of OSCC risk assessment following surgical excision (such as lesion recurrence, number of recurrences, and surgery modality) may be required for models intended to predict oral cancer postoperatively^58^.

In addition, as OPMDs represent a heterogeneous condition with different clinical subtypes, the exact disease entities in which risk stratification may be beneficial using the AI platform should be made known^1^. Of note, reference should be made to the exact definition and surgical diagnostic approach of the OPMD subtypes intended for AI-based risk stratification to be able to realize similar performances in surgical centers intending to validate and apply the AI platforms available.

### AI Model construction should be geared toward clinical application rather than ‘feasibility experiments’

The majority of AI-based models for perioperative cancer risk assessment in OPMDs were implemented using small sample datasets from single treatment centers without independent or external validation. This implies that the aim of developing such models might be to assess the feasibility of AI algorithms in predicting malignant transformation among OPMD patients. As there is the need for a paradigm shift towards constructing robust models that are streamlined for clinical application, as a refinement, it should become necessary that AI feasibility experiments and models constructed with low-volume datasets be limited to the assessment of the performance of novel AI architecture for OSCC risk prediction by transfer learning^60,61^. Additionally, small cohort sizes should be used for AI-based model construction with the hope of retraining when more datasets are available in the future^28^. Emphasis should be made on the construction of models using large multinational cohorts of patients with OPMDs across different surgical teams and centers and multiple rounds of external validation with the performance measures of the AI-based models compared to those of the OED grading system.

### Stratify performance of AI model/platforms by OPMD subtype and dysplasia status

The clinical entities of OPMDs exhibit a distinct malignant transformation rate^1,2^. PVL and erythroplakia have the highest malignant transformation proportion that ranges from 26.7 to 72.4% and 13.6 to 56.1%, respectively, followed by leukoplakia (5.9–14%), oral submucous fibrosis (2.9–8%), oral lichenoid lesions (1.6–7%), and oral lichen planus (0.9–2.3%)^30,62–66^. While OPMDs with an increased likelihood of OSCC development often warrant surgical intervention, risk stratification is required to determine the need for surgery among patients with OPMDs that bear low or equivocal malignant transformation rates (e.g. leukoplakia and lichenoid disease). Hence, OPMDs should not be categorized as ‘one disease’ during AI model development. Preferably, AI model construction and validation should be streamlined to a single OPMD subtype or a few OPMDs that share the same predictive factors. If disparate OPMD subtypes are used to construct AI models, the validation performance (discrimination and calibration) should be investigated for each clinical subtype to assess whether the intelligent model performs satisfactorily for all subtypes.

Epithelial dysplasia often represents one of the most important determinants of malignant transformation among patients with OPMDs^11^. Often, OPMD patients with OED have an increased probability of developing OSCC than those without dysplasia^3,12^. As a result, stratifying the dysplasia status and assessing whether potential benefit exists in employing AI-based risk stratification among those with dysplasia is crucial to understanding whether AI can further identify those at the ‘highest risk’ of developing OSCC beyond the limits of OED assessment. Ultimately, performing subgroup analysis by clinical subtype and OED status to comprehensively determine the validity of AI-based tools for perioperative cancer risk prediction in OPMDs will help unravel potential patient subgroups in which the AI model or platform may have suboptimal performance thereby streamlining the application of the models in surgical centers during full-scale implementation.

### Performance evaluation of AI-based models needs improvement

Metrics that have been employed to determine the performance of AI models for malignant transformation prediction in OPMDs largely assess their discriminative ability. The calibration and stability of these models have been infrequently assessed. Given that a significantly lower proportion of patients with OPMDs will develop OSCC, there is a need to underscore the use of predicted probabilities rather than outright binary classification of malignant transformation risk during perioperative cancer risk stratification^30^. As such, visualization techniques such as plotting reliability plots or estimates such as the Brier score and integrated calibration index may be employed to assess the calibration and robustness of predicted risk probabilities in future AI-based models^67^. Likewise, correlations, SD, and coefficient of variations of discriminatory and calibration performance metrics across cross-validation folds or bootstrapped samples may be used as a measure of model stability for AI models upon training^54,58,68,69^.

### Net benefit analysis of AI-based models is mandatory

Discriminative and calibration measures of AI model performance may not address the issue of potential clinical benefits or risks involved if models are employed to support the selection of patients that would require surgical excision^70^. The fact that AI models may outperform dysplasia grading in terms of accuracy does not mean that additional benefits may be realized if implemented. Therefore, the net benefit of intelligent models should always be determined in addition to traditional performance metrics using methods such as decision curve analysis (DCA) and clinical impact plots^70,71^. Of note, DCA analysis to evaluate clinical net benefit was performed for only one intelligent model proposed so far for perioperative OSCC risk stratification in OPMDs^58^. DCA plots at feasible threshold probabilities (usually below 40–50%) will help determine whether superior benefit exists in selecting only patients deemed to be high risk and require surgery based on AI risk stratification in comparison to when all patients undergo surgical intervention. Moreover, the net benefits of AI models can be compared to other statistical models and OED grading systems.

### Molecular biomarkers may improve the precision of AI-based platforms

It is common for studies reporting the construction or validation of intelligent models for risk stratification of OPMDs to cite the absence of molecular markers in their predictive factors as a limitation of their studies. Several biomarkers have been identified to predict malignant transformation in disparate OPMDs including loss of heterozygosity (LOH), tissue-based molecular markers (e.g. p53, ALDH1, CA9, Ki-67, PD-L1, and S100A7), mRNA expression, and methylome biomarkers^15,59,72–75^. The future should see the direct interrogation of these molecular features using conventional machine learning algorithms and neural networks to determine their potential application independently. Moreover, since the present AI platforms and models for OPMD risk stratification have displayed poor precision, integrative models constructed based on conventional (clinical or histologic) features and molecular markers may help mitigate this challenge; thus, supporting individualized risk stratification and treatment planning.

### AI-based platforms for OSCC risk assessment in OPMDs should be easily accessible

Since AI-based risk stratification in OPMDs is presently not part of standard care in surgical practice, intelligent platforms should be presented as accessible and free interactive platforms such as web tools or applications^76,77^. Increased accessibility of the tools will make them attractive to other surgical centers for further validation and application. Likewise, this will allay concerns about the potential increase in the cost of care that employing these AI tools may pose and help prevent this as a barrier to implementation in surgical practice.

### AI-based platforms should be externally validated locally before clinical implementation

Given that three AI platforms exist currently^54,58^, and more models will become available soon for perioperative OSCC risk stratification of OPMDs, it is sacrosanct to assess the performance of any AI tool using some data available in local institutions before full-scale clinical application. One key issue with intelligent platforms is that they are as robust as the data employed to construct them and may display limited performance in other environments or clinical scenarios especially if the training dataset is limited^27,28^. Cross-institutional generalizability evaluation of the AI-based risk stratification models should first determine the difference in the characteristics and presentation of OPMD cohorts used to construct the platforms and local OPMD patients. Afterward, discriminatory performance metrics including sensitivity, specificity, and precision, and calibration metrics (Brier score and calibration plots) may be determined and compared to those presented in the development cohort. Moreover, the net benefit of the platforms for guiding surgical treatment selection and follow-up decisions of OPMDs should be assessed locally^71^. If found suboptimal, models may be retrained using local data in these surgical practices if codes have been deposited in public repositories or the model may be deemed unfit for use in OPMD risk stratification if local redevelopment is not feasible.

### Improvement in the choice of datasets and AI algorithms for OSCC risk prediction may be necessary

Structured datasets extracted from electronic health records of patients with OPMDs have been largely used to implement AI-based models for cancer risk stratification. This has limited the choice of AI algorithms in OSCC risk prediction to conventional machine learning classifiers like random forest and even precluded the increased application of state-of-the-art neural networks that are capable of providing unbiased predictions without limiting the flexibility of dataset use. Deep learning algorithms are likely to eliminate manual feature extraction of unstructured data and have better discriminatory performance than conventional models especially when trained with a large volume of data^78^. As such, more focus should be placed on employing unstructured data including clinical photographs, whole-slide images, spectral images from optical visualization techniques, texts from electronic records, and medical images directly in their native formats. Ultimately, this practice could potentially facilitate the use of robust innovative AI algorithms or frameworks such as Generative Adversarial Networks for data augmentation, self-supervised learning and processing of electronic records and clinical images, transfer learning for image classification, and attention-based multiple instance learning for whole-slide histopathology images in the perioperative cancer risk assessment of OPMDs^60,61,79–81^. Also, implementing intelligent platforms for OSCC risk stratification with unstructured data is likely to eliminate interobserver biases associated with the diagnosis and assessment of key features in OPMDs such as OED grading and clinical description of lesions^11^.

### Data quality should be evaluated before model construction

Assessment of data quality was not intentionally performed or documented for any of the AI models for perioperative cancer risk stratification, which mirrors the practice of AI model development for head and neck cancer. To aid the construction of data-centric intelligent platforms with the potential for clinical implementation, structured, and unstructured datasets should undergo quality checks before model training^82^. Knowledge of data quality may prompt the use of modifications and appropriate preprocessing techniques to adjust shortcomings in the datasets of patients with OPMDs before model training. Structured datasets may be evaluated for completeness, fairness, outliers, target class balance and representativeness, label purity, collinearity, feature relevance, and class overlap^28,29^. In addition, for unstructured datasets, resolution and clarity should be evaluated along with other feasible data quality assessment parameters.

## Conclusions

AI can potentially offer better risk stratification in the surgical management of patients with OPMDs than the current method of dysplasia assessment and grading. However, AI models or platforms developed for this purpose are not streamlined enough for clinical application in surgical practice currently. Above, this review has presented a strict implementation pathway, discussed challenges to implementation, and highlighted specific areas for refinement to guide the development, validation, deployment, and clinical application for future AI methods aiming to predict malignant transformation in OPMDs to determine the need for surgical intervention. Hopefully, this drives the field a step closer to achieving a consensus AI tool/platform that improves the surgical management of patients with OPMDs for better prevention and early detection of oral cancer.

## Ethical approval

Not applicable.

## Consent

Not applicable to this study.

## Sources of funding

This study was supported by the Hong Kong Research Grants Council General Research Fund (Project No 17117523). The funders are not involved in the design, writing, and implementation of this study.

## Author contribution

J.A.: was involved in study concepts, data curation, visualization, writing original draft and reviewing and editing of the article; Y.-X.S.: was involved in the study concepts, funding acquisition, project administration, supervision, and writing review and editing.

## Conflicts of interest disclosure

The authors declare that they have no financial conflict of interest with regard to the content of this report.

## Research registration unique identifying number (UIN)

1. Name of the registry: not applicable.
2. Unique identifying number or registration ID: not applicable.
3. Hyperlink to your specific registration (must be publicly accessible and will be checked): not applicable.

## Guarantor

John Adeoye.

## Data availability statement

None required for this article.

## Provenance and peer review

Not commissioned, externally peer-reviewed.
